# EEG biomarker candidates for the identification of epilepsy

**DOI:** 10.1016/j.cnp.2022.11.004

**Published:** 2022-12-14

**Authors:** Stefano Gallotto, Margitta Seeck

**Affiliations:** EEG and Epilepsy Unit, University Hospitals and Faculty of Medicine, University of Geneva, Rue Gabrielle-Perret-Gentil 4, 1205 Geneva, Switzerland

**Keywords:** Epilepsy, EEG biomarkers, IEDs, Connectivity, Microstates, HFOs

## Abstract

Electroencephalography (EEG) is one of the main pillars used for the diagnosis and study of epilepsy, readily employed after a possible first seizure has occurred. The most established biomarker of epilepsy, in case seizures are not recorded, are interictal epileptiform discharges (IEDs). In clinical practice, however, IEDs are not always present and the EEG may appear completely normal despite an underlying epileptic disorder, often leading to difficulties in the diagnosis of the disease. Thus, finding other biomarkers that reliably predict whether an individual suffers from epilepsy even in the absence of evident epileptic activity would be extremely helpful, since they could allow shortening the period of diagnostic uncertainty and consequently decreasing the risk of seizure. To date only a few EEG features other than IEDs seem to be promising candidates able to distinguish between epilepsy, i.e. > 60 % risk of recurrent seizures, or other (pathological) conditions. The aim of this narrative review is to provide an overview of the EEG-based biomarker candidates for epilepsy and the techniques employed for their identification.

## Highlights

• Reliable epilepsy biomarkers would be extremely helpful for patient diagnosis and treatment.
• Several EEG features are promising biomarker candidates.
• Recent methodologies hold great potential for the discovery of new EEG biomarkers.

## 1 Introduction

Epilepsy commonly shows the recurrent presence of either focal (partial) or generalized seizures (Sazgar and Young, 2019, Thurman et al., 2011). Focal seizures refer to abnormal activity in one or more circumscribed regions of the brain, whilst generalized seizures occur when this abnormal activity affects both hemispheres, encompassing their cortical and subcortical structures (Fisher et al., 2017). On average, this disorder affects 0.5–1 % of the human population (Sander, 2003, Ngugi et al., 2011), with 30–40 % of it becoming pharmacoresistant (Sharma, 2015).

Even though the presence of seizures is one of the main characteristics of epilepsy, their occurrence does not assure the patient will eventually develop the disease (Angus-Leppan, 2014). In fact, about 10 % of individuals may have a single seizure in their lifetime as a symptom of an acute insult of the central nervous system, e.g. severe hyponatremia. As a consequence, it is often difficult for clinicians to provide immediately a correct diagnosis after a first seizure occurs (Krumholz, 2009), with an overall misdiagnosis of epilepsy estimated to be up to 23 % (Ferrie, 2006, Oto, 2017, van Donselaar et al., 2006). On the one hand, this leads to unnecessary medical treatment with undesirable side effects in case the patient does not actually have epilepsy. On the other hand, withholding treatment when it is indicated and leaving patients untreated could be even more crucial since it increases the likelihood of other seizures, accompanied by an increased risk of trauma or, in some cases, mortality (Engel et al., 2018, Hauser, 2014, Kløvgaard et al., 2022).

Given the difficulty of diagnosis of a first seizure, a tool that helps clinicians to identify and differentiate characteristics of brain activity strictly related to epilepsy would be enormously helpful. Being able to identify specific features of the EEG signal that reliably allow detecting epilepsy could lead to fundamental improvements when assisting and treating a patient arriving at the hospital and complaining about a suspected first seizure. Moreover, fast screening tools could provide medical doctors with qualitative and quantitative measures, allowing them to estimate how successful the prescribed treatment will be. Ideally, this tool should have the ability to:

• distinguish reliably between a first epileptic event in the context of an early-onset epilepsy disorder and a non-epileptic event, or an acute symptomatic seizure due to a transient systemic insult
• identify, in case of a confirmed epileptic disorder, the type of epilepsy (e.g. generalized or focal), as prognosis and treatment differ between epileptic disorder categories
• be easily usable in a fully automatic manner, thus lifting the workload from medical doctors who would then obtain reliable results simply by feeding the EEG signal into a dedicated software

Recent studies have demonstrated the possibility to assess the presence of epilepsy by looking at different measures, making researchers think these could be reliable biomarkers allowing its detection and diagnosis. These biomarker candidates span from gene expression (Weber et al., 2014, Rakhade et al., 2007), metabolic biomarkers (Berg et al., 2010), changes of the structure (Sloviter and Bumanglag, 2013, Fahoum et al., 2012) and function (Carboni et al., 2019, Carboni et al., 2020) of the brain.

Electroencephalography is the most accessible and established technique in the study of epilepsy. Thus, in this review we will discuss how EEG is clinically applied in this context, as well as the established or potential electrophysiological signal changes that could be reliable EEG biomarkers of epilepsy. These changes can be related to specific aspects of the disease, depending on whether there is a high risk of seizures to occur (interictal state) or seizures are occurring (ictal state). In clinical practice, the doctor is almost always confronted with an EEG obtained during the interictal state, with the EEG tracing not necessarily showing any particular signs attributable to the disease. It becomes therefore evident that the identification of epilepsy also in the absence of seizures is of paramount importance. However, hitherto no EEG biomarker has been identified as being highly reliable in detecting and predicting epilepsy (Engel, 2013), in particular in the interictal phase. In the next sections we will review potential EEG biomarkers of epilepsy, and describe the most recent methods employed to extract them from the EEG signal.

## 2 EEG biomarkers

A *diagnostic* biomarker should be able to accurately detect in a reproducible manner the presence of a certain disease and distinguish between its subtypes (Strimbu and Tavel, 2010, Califf, 2018). Epilepsy is considered both a structural and a functional network disorder (Richardson, 2012, Kramer and Cash, 2012) involving regions interconnected locally and remotely across the brain (Carboni et al., 2019). Thus, when attempting to identify biomarkers, previous studies have tried to investigate abnormalities in the structure as well as the functional network properties of the brain. While differences in the structure of the brain between patients and healthy controls may be found regardless of the presence of epileptic events, these differences become more difficult to detect when brain activity is investigated. Here, we review different electrophysiological biomarkers for the identification of epilepsy when the disease has already manifested.

### 2.1 Current EEG biomarker

#### 2.1.1 Interictal epileptiform discharges

**Fig. 1:**
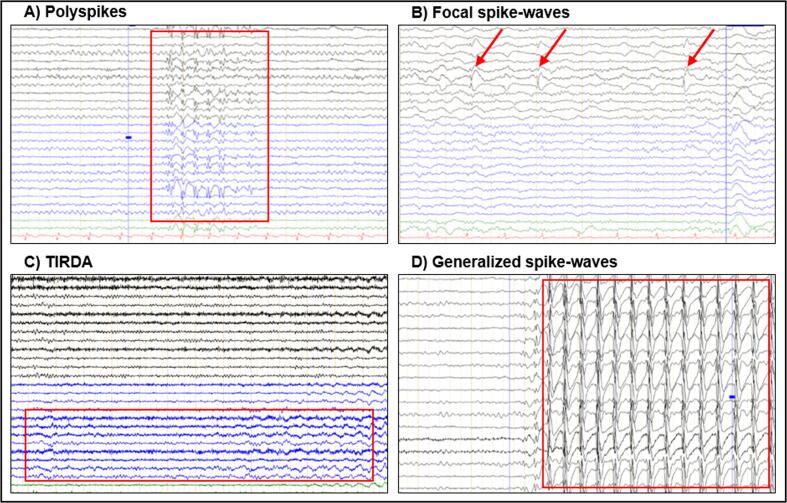
Examples of A) polyspikes, B) focal spike-waves, C) temporal intermittent rhythmic delta activity (TIRDA), and D) generalized spike-waves. Red rectangles and arrows indicate EEG patterns of interest. (For interpretation of the references to colour in this figure legend, the reader is referred to the web version of this article.)

Among all EEG biomarkers of epilepsy proposed over the years, interical epileptic discharges (IEDs) are undoubtedly the best known and established biomarker (Engel et al., 2013). IEDs include spikes, polyspikes, and sharp waves (alone or combined with a following slow wave). More recently it has been shown that focal or lateralized intermittent rhythmic delta activity with monomorphic features is also associated with seizure activity (Gelisse et al., 2011, Gaspard et al., 2013). A large study, including>13,000 airline pilots undergoing routine EEGs as part of their assessment protocol, found IEDs in only 0.5 % of otherwise healthy subjects (Gregory et al., 1993). Most often, IED-patterns in non-epileptic patients are the result of neuroleptic medications (personal observation). Thus, when IEDs are present, they can be considered valid EEG biomarkers of epilepsy, with a sensitivity higher than 90 %. However, while the presence of IEDs likely indicates an underlying epileptic disorder, most EEG tracings of patients with epilepsy are negative, even in case of a very active epilepsy. In fact, in adult first-seizure patients only 12–50 % of routine EEGs show IEDs (Werhahn et al. 2015), with a slightly higher percentage in pediatric EEG due to a more frequent underlying generalized epilepsy (Wirrell, 2010). Fig. 1 includes different types of IEDs, as well as other typical epilepsy patterns.

In patients with a clear diagnosis of epilepsy but a negative routine EEG, it is assumed that IEDs are present in the EEG tracing but cannot be identified by the epileptologist who is performing the visual reading, thus being defined as “hidden” IEDs. Several factors can lead to this condition: a) the IED is too small and therefore overlooked, b) the IEDs have their origin in brain areas that are deep and cannot be easily detected from scalp electrodes (e.g. insula), or c) that are scarcely covered by scalp electrodes (e.g. basal temporal areas). Depending on which one of these scenarios is the most likely explanation, different methods can be implemented. In the first case, zooming in on the electrodes that most likely cover the possible focus (e.g. left hemispheric contacts in case the patient presented a right eye and head deviation) may increase the chance to identify the IED. In the second and third cases, augmenting the number of electrodes (i.e. using 40–64 electrodes, or more) could lead to a better coverage of the scalp and thus to an increased likelihood of observing the IED.

Several studies evaluated the possibility of identifying “hidden” IEDs from the EEG signal in the time domain, frequency domain, and time–frequency domain. These methods have been further explored in terms of relative amplitudes, slope, and curvature. Chavakula et al. (2013) used a wavelet transform-based algorithm directly applied to the raw EEG data and compared accuracy in five different conditions: (1) by taking in consideration only one channel, or (2) multiple channels across different sleep-wake cycles, (3) by testing accuracy in identifying left vs right hemispheric IEDs, (4) by applying machine learning (ML) to improve detection performance, (5) over all sleep-wake cycles or only during wakefulness. The authors showed that their algorithm was largely insensitive to artifacts and able to rapidly and accurately quantify and localize interictal spikes directly from the unprocessed EEG signal, both during wakefulness and sleep (sensitivity > 70 %, specificity > 80 %). Thomas et al. (2018) analyzed 30-minute EEG recordings of 156 patients with epilepsy (93 EEGs with marked IEDs and 63 IED-free EEGs) in combination with an EEG classification system which included three modules (pre-processing, waveform classification, and EEG classification). The authors down-sampled the data to 128 Hz, applied a notch filter at 60 Hz, and a 1-Hz high-pass filter to remove baseline fluctuations. They then classified specific waveforms into either IEDs or background activity. The output generated by this classification was further analyzed applying a ML algorithm which eventually led to an accuracy in IED detection of 84 %.

While these methods require deep methodological knowledge, simpler parameters can also be very useful when it comes to IED identification, as recently demonstrated by Palepu et al. (2018). The authors set different thresholds at which the level of brain activity would be considered a spike. Threshold values (amplitudes) from −0.3 to −0.7 in increments of 0.02 were chosen to ensure that the optimal threshold was captured within this range. These thresholds were then tested in order to identify which one performed best in identifying real spikes. The most accurate results were observed using a threshold of −0.46, leading to a true positive IED detection rate of 98 % and a false positive rate of 2 % approximately, almost matching the performance obtained by a board-certified EEG reader. IEDs are undoubtedly-one of the main EEG features able to diagnose and monitor epilepsy, with certain spike patterns being specific to distinct epilepsy syndromes (e.g. generalized three-*per*-second spike-and-wave of absence epilepsy), thus allowing the diagnosis of the syndrome and the optimal choice of drug treatment. Given that a negative EEG is not equal to the absence of epilepsy, however, the correct diagnosis (epilepsy yes/no) remains a particularly challenging task.

### 2.2 EEG biomarker candidates

#### 2.2.1 High-frequency oscillations

High-frequency oscillations (HFOs) are brief oscillatory field potentials (80–500 Hz) that were recorded for the first time in the hippocampus of rats and in the entorhinal cortex of humans more than 20 years ago (Bragin et al., 1999, Buzsáki et al., 1992). Two types of HFOs (also defined as ripples) have been described: physiological HFOs (80–250 Hz) and fast HFOs (250–500 Hz). Physiological HFOs occur during sleep (Wilson and McNaughton, 1994) and other normal cognitive functions such as, for example, memory processing (Barth, 2003, Kucewicz et al., 2014, Lachner-Piza et al., 2021). Fast HFOs have been associated with epilepsy, and numerous animal and human studies, mainly from intracranial recordings, were conducted over the years using them to better delineate the seizure onset zone and establish their clinical yield (Staba and Bragin, 2011).

Fast HFOs were found in the epileptic zone (EZ) (Bragin et al., 1999, Liu et al., 2018, Jefferys et al., 2012, Alkawadri et al., 2014) both when epilepsy is still in its early stages and when it has become chronic (Engel et al., 2018, Jacobs et al., 2012), with a few studies reporting that very fast HFOs might even be *exclusively* related to epileptic activity (Usui et al., 2011, Brázdil et al., 2017). Given that IEDs are observed inside as well as outside the EZ in intracranial recordings, with unclear relevance for the surgical success of a possible resection, it has been proposed that fast HFOs could potentially be a more reliable marker for the localization of the EZ (Malinowska et al., 2015, Melani et al., 2013, von Ellenrieder et al., 2016, Jacobs et al., 2012, Zijlmans et al., 2012). However, not many laboratories were able to replicate these early encouraging observations.

**Fig. 2:**
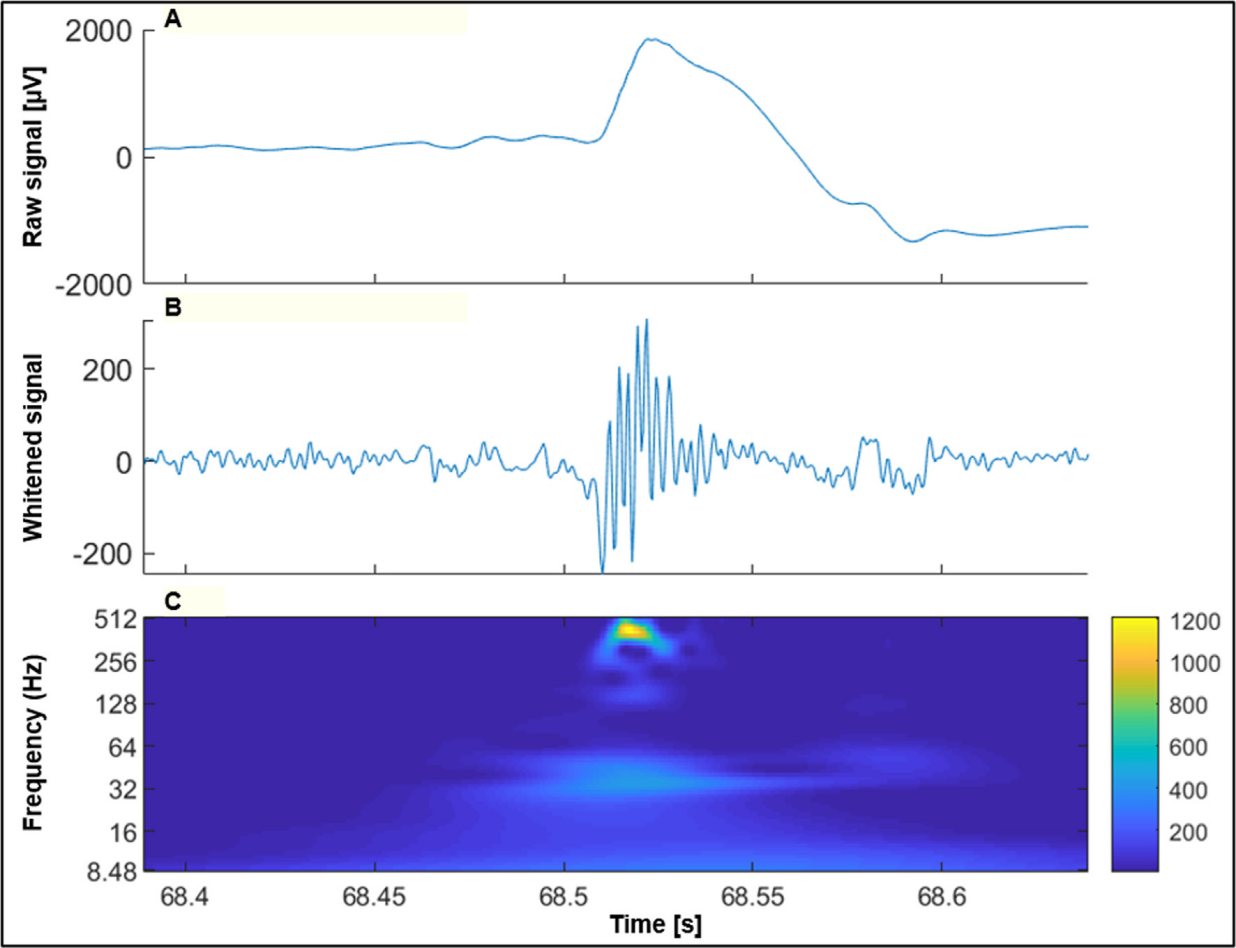
Raw stereo-EEG signal containing a spike-ripple event (A), the same signal transformed (whitened) in order to have a balanced power across frequencies (B), and its time–frequency representation (C).

Spikes and HFOs contain complementary information, and researchers have recently tried to combine both in order to find a superior and more reliable biomarker of epilepsy than either of them alone. A study conducted by Kramer et al. (2019) compared spike-ripple rates between children with benign centro-temporal epilepsy presenting an active disease (<1 year from the last seizure), after a prolonged seizure-free period (>1 year), and healthy subjects. Using (semi-)automated detection techniques, the authors found higher spike-ripple rates in children with active epilepsy both compared to seizure-free children and healthy controls. This result was in line with an earlier observation in children with rolandic epilepsy (van Klink et al. 2016), which showed that ripples co-occurring with spikes indicate a more severe disease than having only rolandic spikes. In adults, the co-occurrence of ripples with spikes seems to have pathological significance, because it delineates more precisely the EZ than IEDs or HFOs alone, as demonstrated in patients with temporal lobe epilepsy (Cai et al., 2021, Melani et al., 2013, Andrade-Valenca et al., 2011, Wang et al., 2013). In line with this idea, Lachner-Piza et al. (2020) developed an automatic detector using machine learning techniques in order to simultaneously identify both biomarkers, and revealed high accuracy in localizing EZs. In Fig. 2 an example of a spike-ripple event is shown.

In a recent prospective study on first seizure events conducted on 56 children (26 presenting epilepsy), the rates of spikes, ripples and spike-ripples were compared in epileptic vs non-epileptic patients. Among the three measures, ripple rates appeared to be the most predictive value, with a sensitivity of 87 % and a specificity of 85 %. Moreover, the authors found a negative correlation with delay of diagnosis and second seizure occurrence (i.e. the higher the rates, the more recent the epilepsy diagnosis and the more likelihood of a second seizure). In this case the authors concluded that in first unprovoked seizures, ripples are the best predictor for the development of epilepsy as compared to spikes and spike-ripples, suggesting their potential role as biomarkers (Klotz et al., 2021).

Despite promising results, recent studies question the unique role of HFOs showing that their ability to identify the EZ is similar to spikes (Lachner-Piza et al., 2020, Roehri et al., 2018) and in contrast to IEDs they rather reflect superficial foci, which could limit their clinical usefulness (Cuello-Oderiz et al., 2017). Moreover, as previously mentioned, HFOs appear to be associated also with normal cognitive processing (Barth, 2003, Kucewicz et al., 2014, Lachner-Piza et al., 2021) and have been found in patients who do not suffer from epilepsy (Gliske et al., 2018, Gliske et al., 2020).

**Fig. 3:**
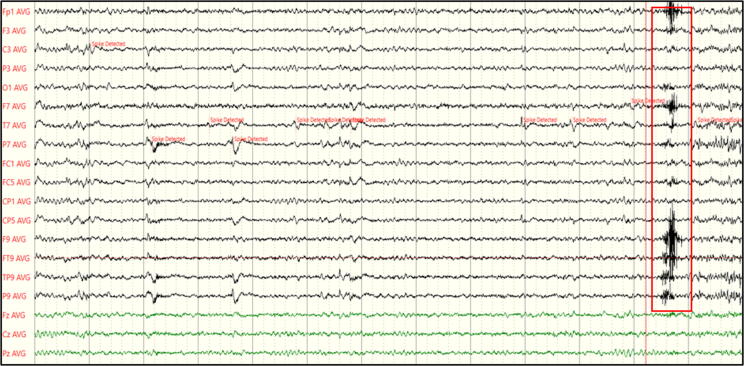
Scalp EEG signal showing a HFO pattern over left fronto-temporal areas (sampling rate 256 Hz).

Besides these concerns, the identification of HFOs also faces many practical challenges (Roehri et al., 2017). Their detection on the scalp EEG is much more difficult than detecting interictal spikes since it requires a markedly higher sampling rate (∼1000 Hz or more), beyond the most commonly used sampling rates of 128–256 Hz. Given the relatively small amplitude and the focality of HFOs, a higher electrode count than the 25 scalp electrodes recommended in clinical routine conditions (Seeck et al, 2017) would also be needed, but since their identification mainly relies on the visual inspection of the EEG tracing, a high number of channels would make the analysis quite arduous. Indeed, recent advancements allowed the development of automatic detector algorithms (Roehri et al., 2016), but these are usually used only as a first step during the detection process, which then still requires a visual confirmation by expert clinicians (Zelmann et al., 2010, Andrade-Valenca et al., 2011, von Ellenrieder et al., 2016). Lastly, various artefacts created by movements or muscle activity can be misinterpreted as HFOs, creating additional difficulties in their classification (Reva and Aftanas, 2004, Yuval-Greenberg et al., 2008, Andrade-Valenca et al., 2011), and hampering their translation as biomarkers into clinical practice. Fig. 3 shows an EEG signal presumably containing a HFO. When recorded from the scalp with a sampling rate of 256 Hz like in this case, the event is not always well recognizable.

In summary, HFOs became of major interest as biomarkers over the past years, with a large body of research investigating their yield in epilepsy. Despite these efforts, most clinical results were obtained from human intracranial EEG data, with the ultimate goal of localizing the EZ in patients with chronic epilepsy (Frauscher et al., 2017, Gerner et al., 2020, Boran et al., 2019). Their clinical usefulness as epilepsy biomarker in scalp EEG is yet to be achieved, also considering that there is no consensus on the classification of pathological and physiological HFOs and the differentiation between them is not always clear (Cimbalnik et al., 2018, Matsumoto et al., 2013, Bragin et al., 2010, Engel et al., 2009, Worrell and Gotman, 2011).

#### 2.2.2 Brain connectivity

**Fig. 4:**
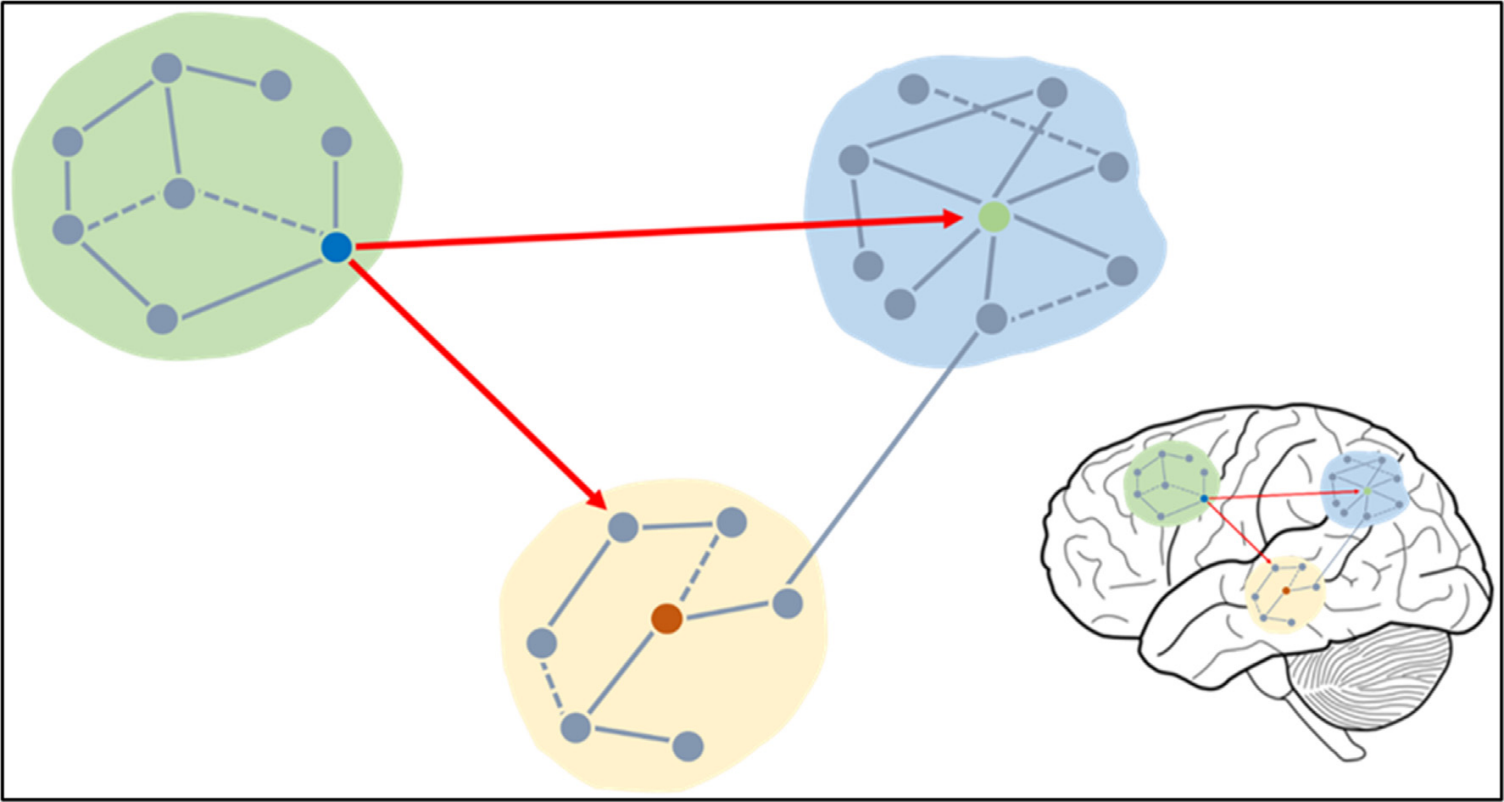
A schematic representation of the modular organization of the brain. Nodes (basic units of a network, represented by the grey circles) and edges (links between basic units, represented by the grey lines) form modules (light yellow, green, and blue backgrounds) and brain networks which communicate with each other. When a node is densely interconnected with other nodes either of the same network or of other networks it is defined as a hub (green, red and blue circles), and can be characterized by different degrees of inter- and intra-modular connectivity. Brain connectivity can be defined as structural, functional and effective connectivity. Structural connectivity highlights anatomical connections between different brain regions (dashed lines), functional connectivity is defined by measures of e.g. correlation between the activity of different brain regions (solid lines), and effective connectivity reveals which node of a given network (driver) exerts a certain influence over another node (receiver) and it is estimated by measures of e.g. granger causality (red arrows). (For interpretation of the references to colour in this figure legend, the reader is referred to the web version of this article.)

In general terms, brain connectivity can be divided into three different categories: structural, functional, and effective connectivity. Structural connectivity is often investigated by means of diffusion-weighted imaging, which is able to identify fibres constituting the anatomical connections between brain regions. Functional connectivity refers to the communication taking place between two given brain regions (measured as statistical dependencies) and can be used as an index to identify a possible underlying disease. Effective connectivity elucidates which one of these given brain regions (called driver) passes the information to the second region (called receiver). Regarding functional and effective connectivity, several instruments can be used, spanning from functional magnetic resonance imaging (fMRI) to EEG and magnetoencephalography (MEG) (Friston, 2011). Fig. 4 shows a schematic representation of these different types of connectivity.

**Fig. 5:**
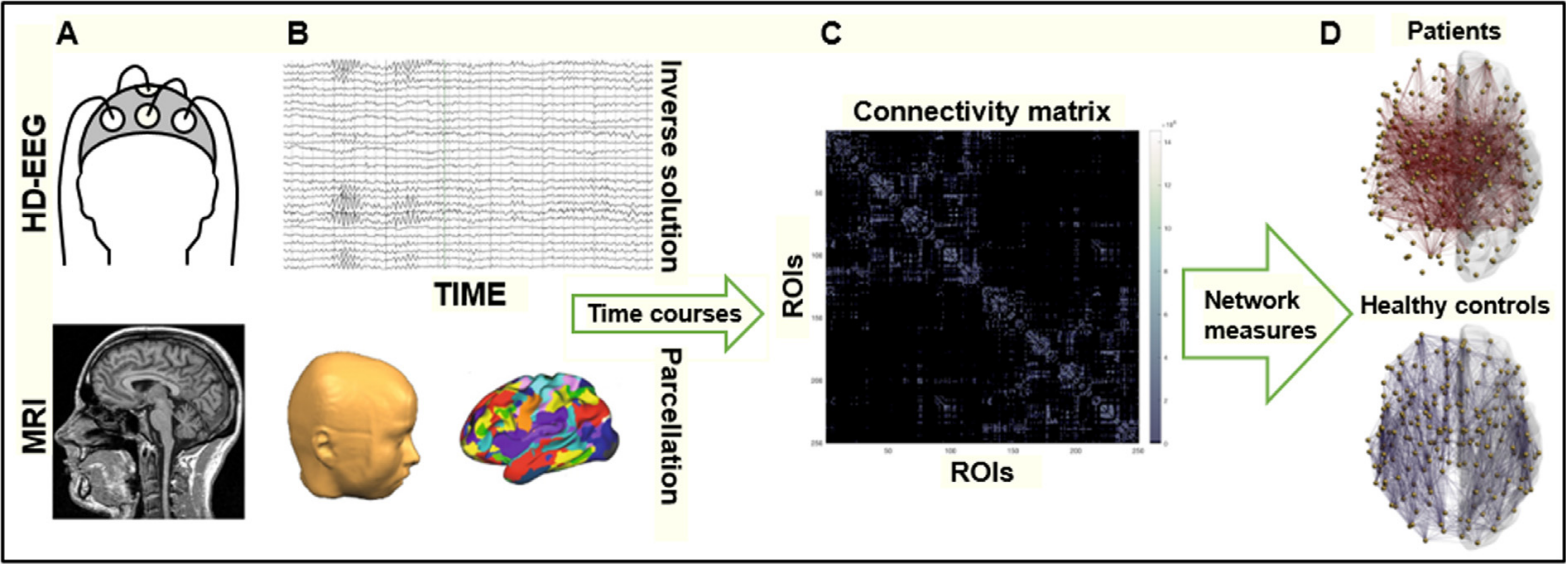
A) EEG and MRI recordings are performed both on healthy controls and epileptic patients. Signals are pre-processed and the result constitutes the input used for source connectivity analyses. B) EEG electrode positions and MRI segmentation are combined in order to estimate source distributions. Brain parcellation leads to a predefined number of regions of interest (ROIs), and for each of them a time course is reconstructed. C) A connectivity matrix representing the pair-wise connections between all ROIs is then computed. D) Nodes and edges constituting brain networks are then analyzed and differences between population groups can be investigated.

Similarly to HFOs, the vast majority of studies on functional and effective connectivity are carried out in patients with chronic epilepsy who are evaluated for surgical treatment. Scalp EEG studies showed alterations of both types of connectivity in the whole brain, but also within specific resting state networks (e.g. attention network), even when the EEG did not show any IEDs (Carboni et al., 2020). In the context of epilepsy, connectivity measures have proven beneficial to differentiate between patients and healthy subjects (Coito et al. 2016), with a higher global efficiency - an index of integration within brain networks - reflecting a pathological functioning of the structures involved (Carboni et al., 2020). Fig. 5 shows an example of a pipeline used for brain connectivity analyses.

Given that EEG is characterized by low spatial resolution, i.e. it is rather difficult to exactly estimate where the signal observed at the scalp level is coming from, connectivity analysis applied to EEG sources represents a promising technique that tries to overcome this issue. In fact, it allows determining the localization of seizure onset zones (van Mierlo et al., 2019, Wilke et al., 2011) as well as prognosis in surgical candidates, even without evident epileptic brain activity patterns (Van Mierlo et al., 2019). As for routine EEG, high- and low-density electrode counts are used, both providing important localizing information (Baroumand et al., 2018, Rikir et al., 2014, Staljanssens et al., 2017). Alterations in brain connectivity can also be identified in an automatic manner, as shown by Verhoeven et al. (2017). The authors were able to classify healthy controls, chronic right and left temporal lobe epilepsy (TLE) patients by investigating EEG epochs without interictal spikes, and estimating directed functional connectivity in three different frequency bands in an automatic fashion (theta, alpha, and beta).

Little is known about whether those changes are already present at epilepsy onset or they occur when the disease is already developed. Ricci et al. (2021) investigated patients with an early onset temporal lobe epilepsy hypothesizing that the efficacy of antiepileptic drug administration could be predicted by connectivity values based on phase locking, an established parameter to describe synchronization between electrodes (or regions). 25 healthy controls were compared to 23 patients at baseline before drug administration (levetiracetam), and three months later. Patients were also compared based on the clinical outcome (seizure-free and non-seizure-free). Lower functional connectivity was observed in seizure-free patients compared to patients who continued presenting seizures after therapy onset. Moreover, a theta power decrease was observed after drug administration compared to baseline and controls, reflecting the direct effect of the drug. The authors concluded that changes in these parameters could predict treatment efficacy with an accuracy up to 91.3 % at baseline and to 86.9 % after three months from the first drug administration. If confirmed in a larger patient population, EEG could play a major role in the prediction to drug response, requiring closer clinical and EEG monitoring in susceptible patients. Up to now, there are no longitudinal studies beyond three months that could determine if continuous drug treatments, number or types of seizures (e.g generalized tonic-clonic seizures) predominantly influence connectivity, perhaps in a permanent fashion.

#### 2.2.3 Microstates

**Fig. 6:**
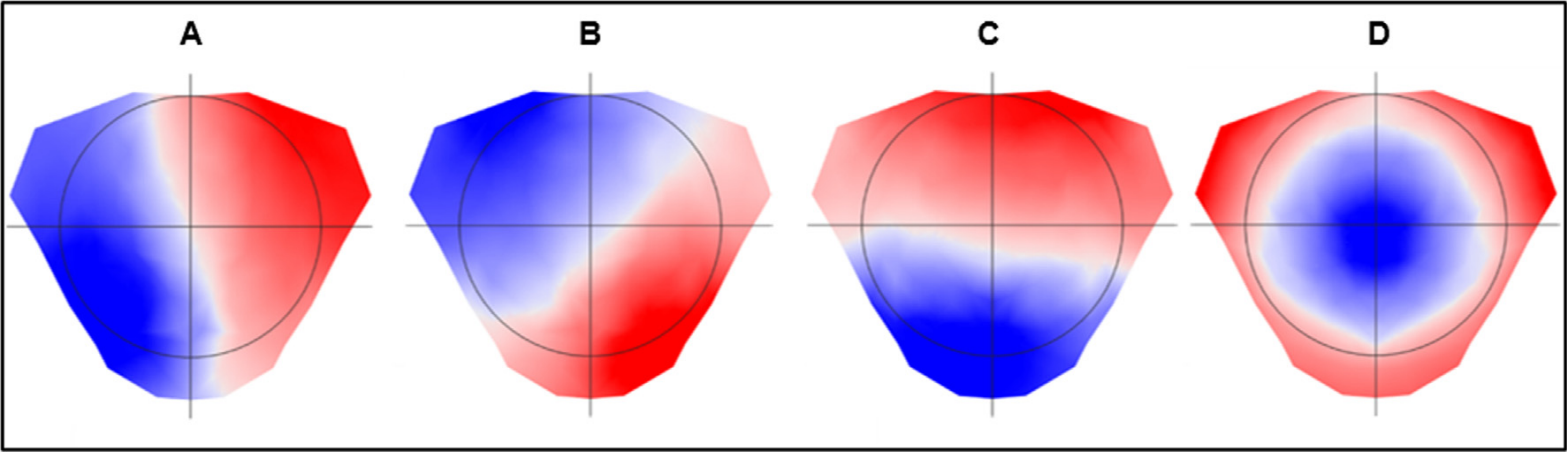
The four most frequently described microstate maps (A, B, C, D). Around 70% of an EEG signal can be explained by a limited number of microstates. These four maps have been repeatedly found in the majority of studies, thus showing high reproducibility and robustness.

Another biomarker candidate for detecting epileptogenic activity in the EEG are the so-called microstates, i.e. topographic maps of the scalp EEG. The concept of microstates originates from the idea that resting-state brain activity is not completely chaotic or random, but instead it can be described by a finite number of maps which change over time, remaining stable only for a short period of time (approximately 50 to 150 ms). Even though several microstate configurations have been found (Khanna et al., 2015, Yuan et al., 2012), most studies showed that four basic microstates explain up to 70 % of the entire EEG data (see Fig. 6; Michel and König, 2018). As these topographic maps offer a millisecond time resolution, they are potentially suitable for detecting rapid changes in large-scale brain networks, giving insights into functional states as well as their aberrations in distinct patient groups (Ahmadi, et al., 2020). For example, comparing healthy controls to patients with absence epilepsy, Liu et al. (2018) demonstrated that the time needed for switching from one basic map to the next was significantly longer in the patient group, suggesting that the duration of the maps could be a biomarker of identifying the disease.

In the study of epilepsy we can broadly categorize microstates into two different classes: the first class relates to the epileptogenic activity itself (“spike-map”), the second class relates to physiological activity during resting state, i.e. with patients being awake with their eyes closed. Baldini et al. (2020) were able to detect spike-maps related to individual IEDs in 25 patients with temporal and extratemporal lobe chronic epilepsy. In EEGs without visually evident discharges, these spike-maps were found abundantly and more frequently in all but 2 patients, compared to 48 controls. In these 2 patients, the spike-maps resembled a physiological microstate, which hampered their distinction as pathological maps. Spike-maps reflecting focal epilepsy invisible to the human eye have also been investigated by a simultaneous fMRI-EEG study including 18 pharmacoresistant patients who later were successfully operated or implanted (Grouiller et al., 2011). The aim of the study was to use time courses (obtained from a correlation between previously acquired long-term clinical monitoring EEG spike-maps and EEG recordings acquired in the scanner) as regressors for fMRI signals related to epileptic activity, in order to better localize the epileptic focus. The authors showed that when this method was applied to the (unrevealing) EEG recordings of the 18 patients inside the MRI, fMRI signals correlating to voltage maps correctly identified the epileptic focus in 14 out of 18 patients. By applying ML, another study showed that neural generators of microstates related to the salience network are altered in patients with temporal lobe epilepsy, and was able to identify patients and healthy subjects with a 76.1 % accuracy (85.0 % sensitivity, 66.6 % specificity), even in the absence of visible interictal epileptiform discharges on the EEG tracing (Raj et al. 2018). Again, these studies were conducted in patients with chronic epilepsy, and the diagnosis of TLE was known.

Microstates reflecting spike-maps could be an interesting parameter for monitoring the disease, however, they first require to be identified and defined at individual patient level. Consequently, they are of less value in case of a first diagnosis, considering also that it remains unclear whether they would work in a mixed epilepsy patient population (e.g. with different foci) and in new onset epilepsy with yet an unknown focus. This is not the case for physiological microstates which occur in all subjects and are relatively robust also in sleep or even coma.

## 3 Machine learning for the identification of EEG biomarkers

The dynamics of an epileptic signal are highly complex, involving different regions and networks of the brain, with signal frequency and amplitude varying over time between and even within patients (Iasemidis et al., 2003). In addition, the EEG tracing contains by nature signals that are noisy and non-stationary, which in a clinical context are also influenced by medications and vigilance states. These aspects make the identification of reliable features across patients still a great challenge (Engel, Bragin, and Staba, 2018) for which tailored automated methods could be an effective solution.

In broad terms, ML can be described as “a computer learning from data without being explicitly programmed” (Samuel, 1959, Hastie et al., 2009). Regarding EEG classification, EEG signals are used as input to an automated system that gives as output specific labels indicating which category the EEG signal belongs to (e.g. normal or epileptic brain activity). The process usually consists of feature extraction (the aspect of the EEG signal that is further analyzed since considered as relevant) and classification (the type of analysis that is performed on these features). ML can be conceptually divided into two different categories: supervised and unsupervised learning. In supervised learning a specific algorithm is trained on data that have previously been labeled, e.g. an EEG of known TLE patients presenting unilateral temporal IEDs. This ultimately allows estimating characteristics of the data that have not been labeled. In unsupervised learning an algorithm is trained to discover patterns, subgroups or trends in the data without the need of being previously labeled (Yu et al., 2018). In both cases, specific aspects or features of the data are identified and used to generate functions that ultimately predict certain outputs such as, for example, differentiating between a healthy brain and a brain with a disorder. Using both categories (supervised and unsupervised learning) at the same time is also possible, combining small datasets that have been labeled with large unlabeled datasets in order to generate a model function (also called classifier) and apply it to a more generalizable sample. These functions are numerous and can be of different types, but their discussion is beyond the scope of this review (for a comprehensive overview see Naser, 2021).

In clinics, recognition and classification of epileptic activity in the EEG tracing is usually performed by expert clinicians who visually identify specific patterns attributable to epileptic activity. This procedure is time-consuming, especially in long recordings or when the reader has limited EEG experience. ML algorithms could help in this context, provided that they are able to automatically recognize and classify pathological EEG patterns. By using a supervised-learning paradigm, Fergus et al. (2015) were able to distinguish between normal EEG and seizure pattern, with a sensitivity of 93 % and a specificity of 94 % regardless of where the seizure was originating, thus being able to generalise across different patients. Similarly, in the study of Acharya et al. (2018) the authors reported high accuracy (88.7 %), sensitivity (95 %) and specificity (90 %) for seizure detection and classification into preictal and seizure categories. Using MEG, Soriano et al. (2017) aimed at differentiating between healthy controls and patients presenting either idiopathic generalized epilepsy or frontal focal epilepsy. They applied ML algorithms to four different features of the signal, namely total and relative power spectral densities (i.e. the power distribution present in the signal across frequencies), phase-locking value and phase-lag index, and distinguished between controls and epileptic patients with high accuracy (86 %). Of interest, these differences were found with resting-state interictal data. The feature that performed best was relative power spectra density, demonstrating that even a relatively simple feature can be used to successfully classify epileptic brain activity from non-epileptic brain activity.

Another interesting set of ML methods are the convolutional neural networks (CNNs). Broadly speaking, CNNs are mathematical models that aim at describing the functioning of the brain and are characterized by several layers, each of them including a predefined number of nodes (also called “neurons”). The number of layers composing a CNN defines its depth, whereas the number of nodes in each layer defines its width. Nodes in a specific layer have connections to nodes in the next layer and these connections are represented as specific parameters (also called “weights”). In simple terms, the different layers convert inputs into a more abstract representation, allowing the classification of high dimensional data. Using deep CNNs in combination with epileptic signal classification, and applying them to different frequency bands, Gao et al. (2020) were able to classify with high accuracy (specificity 97 %, sensitivity 92 %) four states of epilepsy (interictal, preictal duration to 30 min, preictal duration to 10 min, and seizure), which may be of interest in large datasets like EEG monitoring. Another study reported accuracy and specificity above 99 % by transforming the EEG signal into an image domain and using CNNs to detect seizures in an automatic manner (Gómez et al., 2020).

ML techniques show a good accuracy in identifying seizures, but their use when seizures are not present is not yet clear. Future research should focus on this aspect, since it would allow predicting whether a person indeed suffers from epilepsy before a second – potentially harmful – seizure occurs.

## 4 Conclusion

The need for EEG biomarkers able to identify the presence of epilepsy in a reliable and swift fashion has become increasingly important over the past years. Seizures and epilepsy occur most frequently in elderly patients, where epilepsy is a relevant comorbidity in a number of neurological diseases. Given the large differential diagnosis in this patient group, and considering an aging society which faces continuous healthcare system budget reductions, rapidity and accuracy are of utmost relevance. IEDs – the only established biomarker of epilepsy so far – are present only in a minority of EEGs. Thus, the discovery of another reliable biomarker (with a sensitivity and specificity > 90 %, considering the prevalence of cases in the clinical population of interest (Pepe et al., 2016)) would help clinicians to diagnose the disorder in the absence of clear epileptic activity, allowing the prescription of an appropriate treatment with minimal delay.

## 5 Limitations

Many features of an EEG signal have been proposed as potential biomarker candidates, with novel techniques such as ML constituting a great potential for their classification. However, being most of the studies conducted in a research environment with minimal noise, comparing healthy vs epileptic subjects in a retrospective and exploratory manner, without blinding and including a low number of participants, when the same methods are applied in clinical setups where such conditions are rarely met, they often fail to reproduce the expected outcomes. All these aspects should be considered when conducting future studies.

## 6 Future perspectives

A biomarker that effectively identifies an underlying epileptic disorder in scalp EEG, if there are no IEDs, still needs to be identified. In this regard, future developments should focus on:

- Large-scale prospective studies of mixed patient populations
- Testing innovative hypotheses and methods in real-life clinical setups
- Reproducibility of the results

## Declaration of interests

Margitta Seeck has shares in Epilog©. SG and MS were supported by the Swiss National Science Foundation (180 365, 163398).

## Declaration of Competing Interest

The authors declare that they have no known competing financial interests or personal relationships that could have appeared to influence the work reported in this paper.
